# The *Tetramorium squaminode* species group (Hymenoptera, Formicidae) in the Arabian Peninsula, with a new record from the Kingdom of Saudi Arabia and keys to Arabian species

**DOI:** 10.3897/zookeys.502.9011

**Published:** 2015-05-04

**Authors:** Mostafa R. Sharaf, Hathal M. Al Dhafer, Abdulrahman S. Aldawood

**Affiliations:** 1 Plant Protection Department, College of Food and Agriculture Sciences, King Saud University, Riyadh 11451, P. O. Box 2460

**Keywords:** Myrmicinae, Middle East, Palearctic Region, Asir Mountains, taxonomy, keys, *squaminode*-group

## Abstract

The Arabian species of the *Tetramorium squaminode*-group are treated. *Tetramorium squaminode* Santschi, 1911 is recorded for the first time from the Kingdom of Saudi Arabia and the Arabian Peninsula. Keys to the two Arabian species of the *Tetramorium squaminode*-group, *Tetramorium latinode* Collingwood & Agosti, 1996 and *Tetramorium squaminode*, based on worker and queen castes, are given and a regional distribution map is provided. Notes on habitats of *Tetramorium squaminode* are presented.

## Introduction

The genus *Tetramorium* Mayr, 1855 is one of the largest and most species-rich ant genera in the Formicidae with 575 described species (www.antwiki.org) distributed worldwide (Brown 2000). Members of the genus are often generalized foragers, known to build nests in leaf-litter, decaying wood, or directly into the soil (Brown 2000). Globally, taxonomic studies have treated the genus in several zoogeographical realms or regions, namely the Afrotropical (Bolton 1976, 1980, 1985; Hita Garcia et al. 2010a, b, c; Hita Garcia and Fisher 2014a, b), Malagasy (Bolton 1979, Hita Garcia and Fisher 2011, 2012, 2014c), Oriental and Indo-Australian (Bolton 1977) and the Palearctic (Csösz et al. 2007; Csösz and Schulz 2010) and for the former Soviet republics (Radchenko 1992a, b).

The majority of the Arabian species of the genus *Tetramorium* can be readily distinguished by the combination of the following characters in the worker caste (Bolton 1994): lateral clypeal portions raised into a sharp ridge or shield in front of the antennal insertions; mandibles armed with 3-4 apical teeth followed by a variable number of denticles; antennae 12-segmented with a 3-segmented club; frontal carinae ranging from well developed (*e.g.* *Tetramorium simillimum*-group) to absent (*e.g.* *Tetramorium caespitum*-group); propodeal spiracles low on the side and distinctly behind the midlength of the sclerite; propodeal spines usually present; sting with an apicodorsal lamellate appendage projecting from the shaft.

The *Tetramorium squaminode*-group was characterized by Bolton (1980): petiole squamiform, higher than long in profile and much broader than long in dorsal view; postpetiole rounded-nodiform; anterior clypeal portion usually indented medially; frontal carinae strongly developed extending back nearly to posterior margin of head; antennal scrobes well developed; petiolar and postpetiolar nodes without sculpture dorsally; standing pilosity usually present and moderately dense. The distribution of species of the *squaminode*-group is mainly restricted to East and/or South Africa but with a single species *sitefrum* Bolton, 1980 in Ghana (Bolton 1980).

The *Tetramorium* fauna of the Arabian Peninsula is still relatively poorly known when taken in consideration of the vastness of the peninsula (3,100,000 km^2^) and many areas are unexplored entomologically. The first contribution to the knowledge of the genus *Tetramorium* of the region was presented by Collingwood (1985) for the Kingdom of Saudi Arabia (KSA), the largest country (2,150,000 km^2^) of the peninsula. He listed thirteen species, one of which, *Tetramorium jizani* Collingwood 1985 of the *simillimum*-group, was described as new. A more comprehensive work treating *Tetramorium* of the Arabian Peninsula was presented by Collingwood and Agosti (1996) reporting sixteen species, two of which were new to science, *Tetramorium latinode* of the *squaminode*-group and *Tetramorium yemene* of the *simillimum*-group, both from Yemen. The invasive species *Tetramorium bicarinatum* (Nylander, 1846) was recorded from the United Arab Emirates (UAE) by Collingwood et al. (1997). Recently, a faunistic list of Formicidae for the UAE was given including six *Tetramorium* species, with *Tetramorium latinode* from Sharjah (Collingwood et al. 2011). Only three invasive species have been reported from the Socotra Archipelago (Yemen), *Tetramorium lanuginosum* Mayr, 1870, *Tetramorium simillimum* (F. Smith, 1851) and *Tetramorium caldarium* (Roger, 1857) (Collingwood et al. 2004). Recently, Sharaf et al. (2012a) described a new species, *Tetramorium amalae* of the *Tetramorium shilohense*-group from the southwestern mountains of KSA based on two workers. Additionally, in the above paper *Tetramorium latinode* was recorded for the first time from KSA, redescribing the worker caste and describing the queen caste for the first time. More recently, Sharaf et al. (2013) reported three *Tetramorium* species from Rawdhat Khorim Nature Preserve near Riyadh, KSA, *Tetramorium chefketi* Forel, 1911, *Tetramorium sericeiventre* Emery, 1877 and the new species, *Tetramorium saudicum* Sharaf, 2013.

In the present study, the species of the *Tetramorium squaminode*-group occurring in the Arabian Peninsula are treated. Two species are known, *Tetramorium latinode* Collingwood & Agosti, 1996 and *Tetramorium squaminode* Santschi, 1911. The latter species is recorded for the first time from the KSA and the entire Arabian Peninsula. Keys to the two Arabian species are provided based on worker and queen castes with notes on habitats of *Tetramorium squaminode* Santschi.

## Material and methods

**Study area.** The Raydah Protectorate in the mountains of southwestern KSA is one of the smallest areas set aside as a conservation reserve area in 1989 and is located 10 km west of the city of Abha, (18°12'N, 42°24'E). The total area is about 9 km^2^, but given a drop of 1000 m in 3 km, the estimated total area is closer to 12 km^2^. The village of Raydah lies at 1600 m just outside the protected area (Newton and Newton 1996).

### Measurements and Indices.

(Following Hita Garcia et al. 2010a)

#### Measurements.

Head length (**HL**) maximum distance from mid-point of anterior clypeal margin to mid-point of posterior margin of head, measured in full-face view.

Head width (**HW**) width of head directly behind eyes measured in full-face view.

Scape length (**SL**) maximum scape length excluding basal condyle and neck.

Eye length (**EL**) maximum diameter of compound eye measured in oblique lateral view.

Pronotal width (**PW**) maximum width of pronotum measured in dorsal view.

Weber’s length (**WL**) diagonal length of mesosoma in lateral view from the postero-ventral margin of propodeal lobe to anterior-most point of pronotal slope, excluding the neck.

Propodeal spine length (**PSL**) in dorsocaudal view, tip of the measured spine, its base, and center of propodeal concavity between spines must all be in focus. Using a dual-axis micrometer spine length is measured from tip of spine to a virtual point at its base where spine axis meets orthogonally with a line leading to median point of the concavity.

Petiolar node length (**PTL**) maximum length of dorsal face of petiolar node measured in dorsal view, excluding peduncle.

Petiolar node height (**PTH**) maximum height of petiolar node measured in lateral view from the highest (median) point of the node to ventral outline. The measuring line is placed in an orthogonal angle to ventral outline of node.

Petiolar node width (**PTW**) maximum width of dorsal face of petiolar node measured in dorsal view.

Postpetiole length (**PPL**) maximum length of postpetiole measured in dorsal view.

Postpetiole height (**PPH**) maximum height of postpetiole measured in lateral view from the highest (median) point of the node to the ventral outline. The measuring line is placed in an orthogonal angle to ventral outline of the node.

Postpetiole width (**PPW**) maximum width of postpetiole measured in dorsal view.

#### Indices

Ocular index (**OI**): EL / HW × 100

Cephalic index (**CI**): HW / HL × 100

Scape index (SI): SL / HW × 100

Propodeal spine index (**PSLI**): PSL / HL × 100

Petiolar node index (**PeNI**): PTW / PW × 100

Lateral petiole index (**LPeI**): PTL / PTH × 100

Dorsal petiole index (**DPeI**): PTW / PTL × 100

Postpetiolar node index (**PpNI**): PTW / PW × 100

Lateral postpetiole index (**LPpI**): PPL / PPH × 100

Dorsal postpetiole index (**DPpI**): PPW / PPL × 100

Postpetiole index (**PPI**): PPW / PTW × 100

### Abbreviations of museums

The collection abbreviations follow Lattke (2000).

CASC California Academy of Sciences, San Francisco, California, U.S.A.

NHMB Naturhistorisches Museum, Basel, Switzerland.

KSMA King Saud University Museum of Arthropods, Plant protection department, College of Food and Agriculture Sciences, King Saud University, Riyadh, Kingdom of Saudi Arabia.

WMLC World Museum Liverpool, Liverpool, U. K.

The map was created by the ArcGIS 9.2 program, with the help of Prof. Mahmoud S. Abdeldayem (King Saud University).

## Results

### Tetramorium latinode

Taxon classificationAnimaliaHymenopteraFormicidae

Collingwood & Agosti, 1996

Tetramorium latinode Collingwood & Agosti, 1996: 335, fig. 12 (w.) YEMEN.
Tetramorium latinode Holotype worker, YEMEN: Ma‘bar, pitfall trap, 11.v.1992 *(M. Mahyoub & A. Drews)* (WMLC).

#### Diagnosis.

**Figures 1–3.**
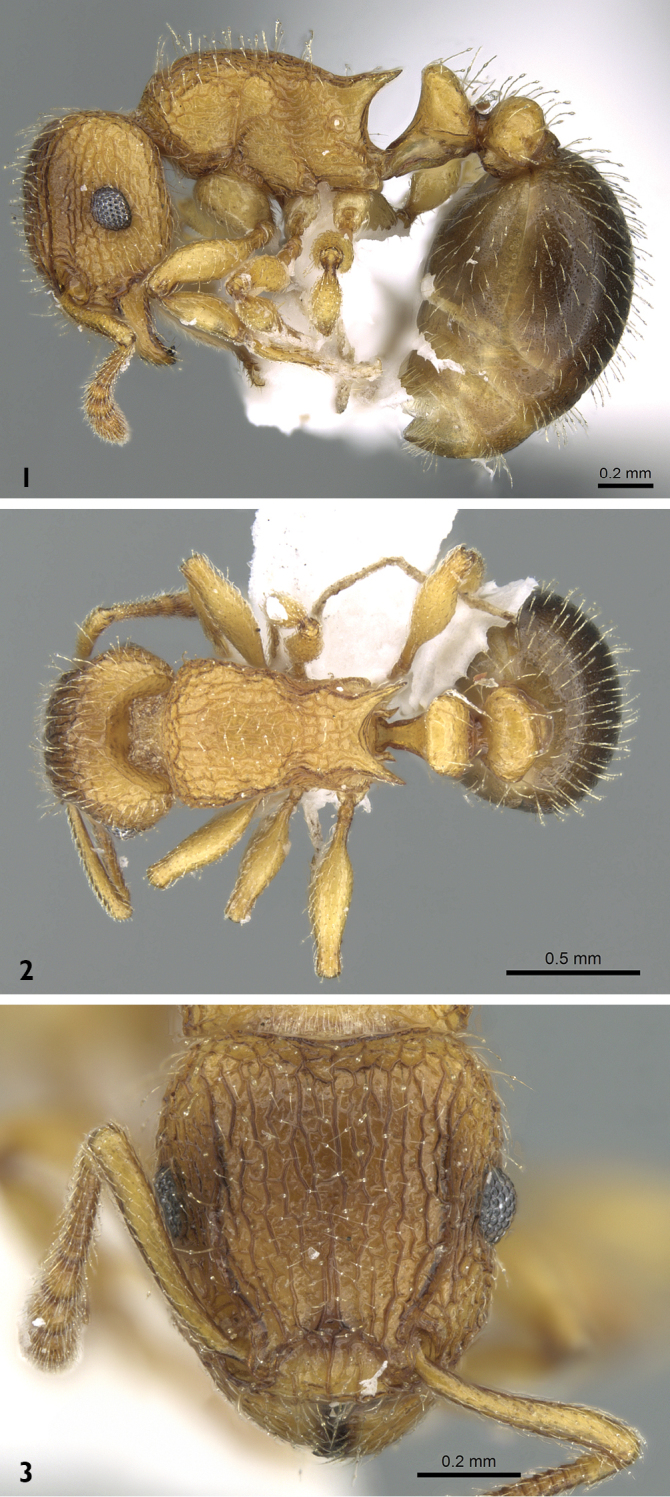
*Tetramorium latinode*, worker (casent0906432, Saudi Arabia). **1** body in profile **2** body in dorsal view **3** head in full-face view. Photographer Estella Ortega, from www.AntWeb.org

**Figures 4–6.**
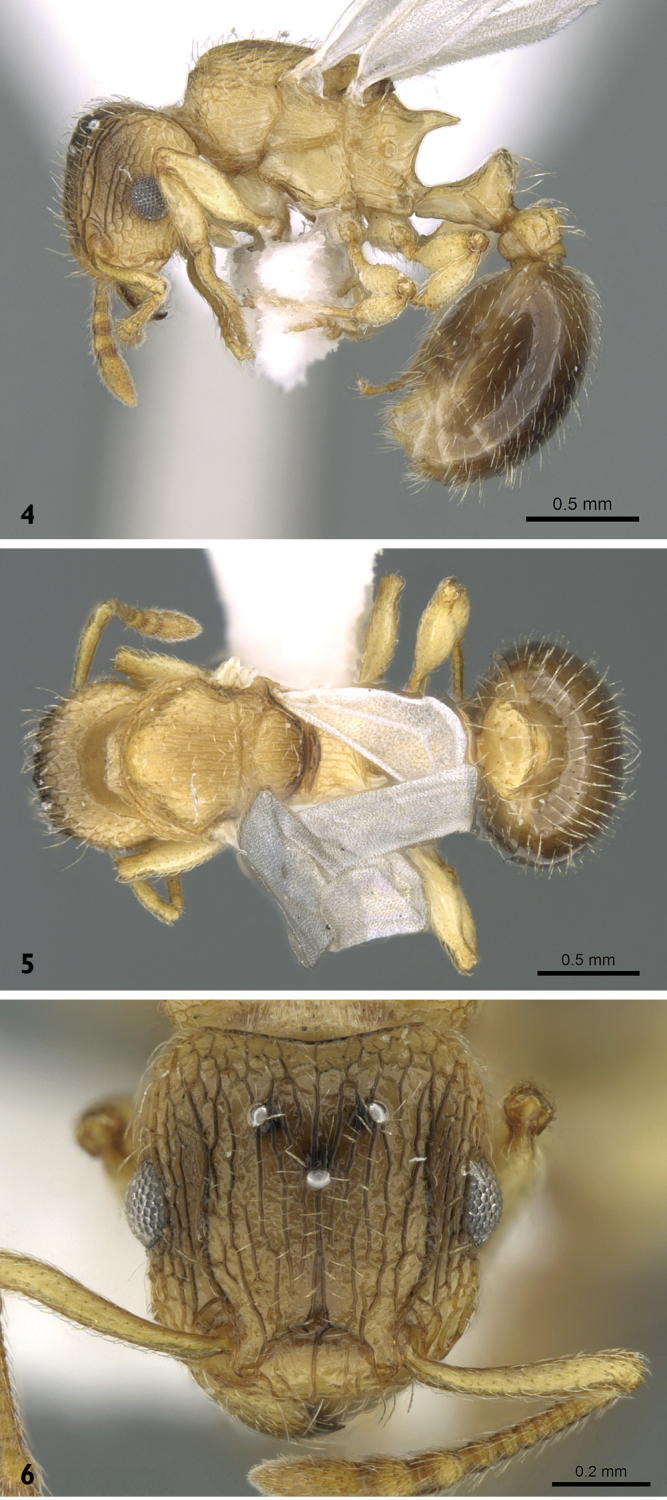
*Tetramorium latinode*, queen (casent0906431, Saudi Arabia). **4** body in profile **5** body in dorsal view **6** head in full-face view. Photographer Estella Ortega, from www.AntWeb.org

***Worker*** (Figs 1–6)**. Head.** Head longer than broad; anterior clypeal margin with a median notch or impression; frontal carinae long and sinuate, reaching back almost to posterior margin of head; antennal scrobes distinct; eyes large (EL 0.12-0.17) with ten ommatidia in longest row. **Mesosoma.** Mesosoma profile a continuous curve; metanotal groove very weakly impressed; propodeal spines long and strong. **Petiole.** Petiole much higher than long in profile, in dorsal view much broader than long but slightly narrower than postpetiole which is clearly broader than long. **Postpetiole.** Postpetiole in profile lower than petiole and broadly rounded. **Sculpture.** Mandibles feebly longitudinally striated; cephalic dorsum and mesosoma irregularly but quite densely longitudinally reticulate-rugulose, petiole dorsum smooth and shining, postpetiole dorsum more or less smooth and shining with vestiges of patchy pattern, gaster smooth and shining. **Pilosity.** Body dorsum densely clothed with long, fine, soft finely barbulate hairs which are fewer on mesosoma and waist; antennae and tibiae with dense decumbent pubescence. Colour yellow, gaster brown to brownish yellow.

#### Material examined.

34 workers, Saudi Arabia, Al Bahah, Amadan forest, Al Mandaq, 20°12.163'N, 41°13.906'E, 1881 m, 19.v.2010 (*M. R. Sharaf & A. S. Aldawood Leg.*); 1 worker, Saudi Arabia, Asir, Abha, Raydah, 18°11.679'N, 42°23.691'E, 1851 m, 8.vi.2014, (Pitfall trap) (*Al Dhafer* et al. *Leg.*); the following materials with data as the previous materials except coordinates and altitudes as follow: 1 worker, 18°11.618'N, 42°23.420'E, 1772 m; 2 workers, 18°11.695'N, 42°23.818'E, 1897 m; 2 workers, 18°12.265'N, 42°24.744'E, 2820 m; 5 workers, 18°12.315'N, 42°24.607'E, 2761 m; 5 workers, 18°12.095'N, 42°24.536'E, 2578 m. (KSMA).

#### Alate gyne.

Head little longer than broad with nearly straight or feebly convex sides; posterior margin of head weakly concave; eyes large and consist of 14 ommatidia in the longest row, EL about 0.27 × HW; antennae 12-segmented; frontal carinae long and sinuate, reaching back almost to posterior margin of head where they merge with the remaining sculpture of cephalic dorsum; antennal scrobes well developed; propodeal spines long and acute; petiole, postpetiole, pilosity and head sculpture as in worker. Bicoloured, body yellowish, gaster brown.

### Tetramorium squaminode

Taxon classificationAnimaliaHymenopteraFormicidae

Santschi, 1911

Tetramorium squaminode Santschi, 1911: 356, fig. (w) Tanzania.
Tetramorium squaminode Santschi 1914: 102 (q.m.). See also: Bolton 1980: 260.

#### Diagnosis.

**Figures 7–9.**
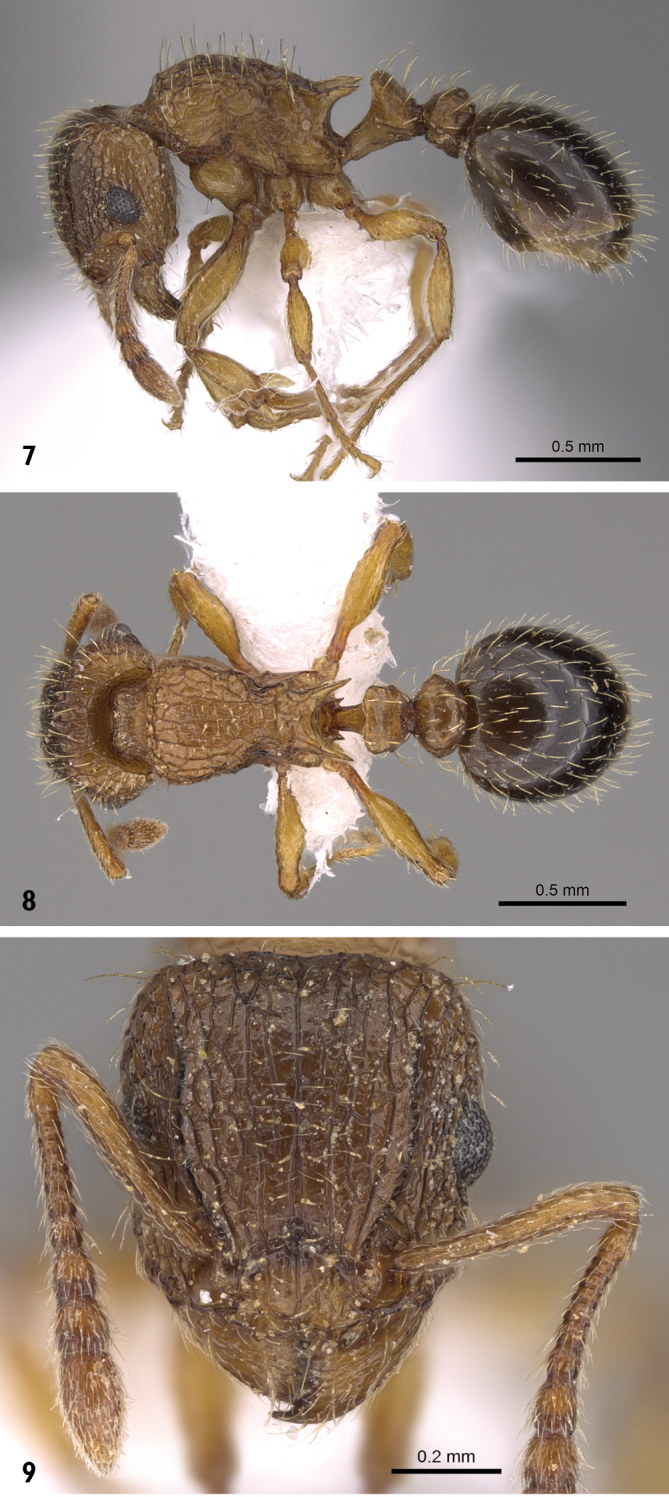
*Tetramorium squaminode*, worker (casent0914585, Saudi Arabia). **7** body in profile **8** body in dorsal view **9** head in full-face view. Photographer Michele Esposito, from www.AntWeb.org

***Worker*** (Figs 7–9). **Head** in full-face view distinctly longer than broad with feebly convex sides and nearly straight posterior margin; anterior clypeal margin superficially concave medially; frontal carinae strongly divergent posteriorly, directed towards posterior corners of head; antennal scrobes well developed; eyes relatively large (OI 22-29) situated on midlength of head, with 13 ommatidia in longest row. **Mesosoma** with rounded pronotal corners when seen from above; in profile metanotal groove feebly impressed or absent; propodeal spines long and sharp; transverse dorsal crest of petiole scale thin and sharp, knife-edged. (DPeI 135-200); in larger specimens petiole dorsum weakly but distinctly emarginate in dorsocaudal view; postpetiole in profile rounded from above; in dorsal view postpetiole hexagonal and much broader than long (DPpI 159–233). **Sculpture.** Cephalic dorsum sharply and irregularly longitudinally rugulose, sculpture running broken from clypeus to posterior margin of head, area in front of eyes strongly reticulate-rugose, lateral sides of head similar but less strongly sculptured; mandibles faintly but distinctly longitudinally rugulose; clypeus with strong median carinae and two pairs of weaker lateral carinae; mandibles faintly but distinctly longitudinally striated; mesosomal dorsum coarsely irregularly reticulate-rugose; area between propodeal spines when seen from above with three transverse carinae; petiole, postpetiole and gaster dorsum more or less smooth and shining, with very faint superficial sculpture. **Pilosity.** Frontal carinae with seven pairs of relatively longer hairs straddling the entire line; posterior corners of head with a single pair of characteristically longer hairs compared to rest of cephalic pilosity which is much shorter; mesosomal pilosity relatively less dense and long, some hairs apically clubbed; petiole and postpetiole with abundant apically clubbed long hairs; legs and antennae with appressed hairs. **Colour.** Cephalic dorsum and gaster dark brown, head sides, mesosoma, petiole and postpetiole yellowish brown.

#### Measurements.

**Worker.** TL 2.70–3.35; HL 0.70–0.75; HW 0.62–0.72; SL 0.42–0.57; EL 0.15–0.20; PW 0.47–0.57; WL 0.75–0.90; PSL 0.15–0.25; PTL 0.15–0.20; PTH 0.27–0.35; PTW 0.25–0.30; PPL 0.15–0.22; PPH 0.22–0.27; PPW 0.32–0.45; CI 84–97; SI 60–81; OI 22–29; DMI 55-67; PSLI 24–35; PeNI 29–60; LPeI 50–67; DPeI 135–200; PpNI 61–90; LPpI 63–100; DPpI 159–233; PPI 117–156 (n=12).

**Figures 10–12.**
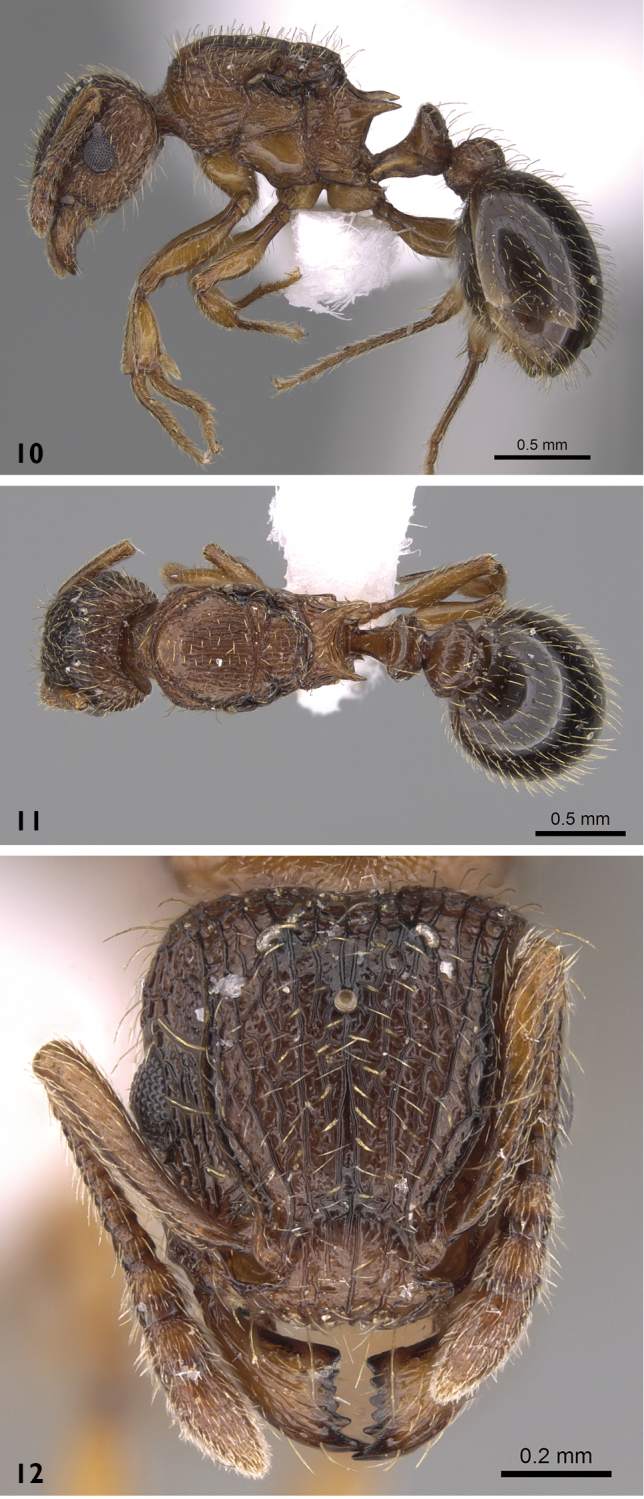
*Tetramorium squaminode*, queen (casent0914584, Saudi Arabia). **10** body in profile **11** body in dorsal view **12** head in full-face view. Photographer Michele Esposito, from www.AntWeb.org

***Queen*** (Figs 10–12)**. Head.** Head clearly longer than broad, narrower anteriorly than posteriorly, with straight posterior margin; median portion of anterior clypeal margin feebly concave; eyes situated nearly at midlength of head; eyes large (OI 28–29) with 16 ommatidia in longest row; antennae when laid back from their insertions fail to reach posterior margin of head. **Mesosoma.** Propodeal spines long thick and sharp; petiole, postpetiole and gaster as in worker caste.

#### Colour.

Head, mesosomal dorsum, petiole and postpetiole dark brown or blackish brown, gaster dark blackish brown to black, antennae and legs yellowish. **Sculpture.** As in worker except mesosomal dorsum longitudinally rugose. **Pilosity.** Pilosity dense over entire body with apically clubbed hairs which are more abundant on postpetiole and rare on gaster; frontal carinae with five pairs of distinctly longer hairs straddling entire length, corners of posterior margin of head with a single pair of longer hairs.

#### Measurements.

**Queen.** TL 3.65–3.75; HL 0.85; HW 0.75–0.80; SL 0.55–0.57; EL 0.22; PW 0.67–0.72; WL 1.12–1.25; PSL 0.20–0.25; PTL 0.20–0.22; PTH 0.40–0.42; PTW 0.35–0.37; PPL 0.20–0.22; PPH 0.32–0.35; PPW 0.45–0.47; CI 88–94; SI 69–76; OI 28–29; DMI 58-60; PSLI 24–29; PeNI 49–55; LPeI 48–55; DPeI 168–175; PpNI 65–67; LPpI 63; DPpI 205–235; PPI 122–134 (n=2).

#### Materials examined.

3 workers, 1 queen, Saudi Arabia, Asir, Abha, Raydah, 18°12.315'N, 42°24.607'E, 2761 m, 26.vii.2014, (Pitfall trap) (*Al Dhafer* et al. *Leg.*); 2 workers Saudi Arabia, Asir, Abha, Raydah, 18°12.095'N, 42°24.536'E, 2578 m; 3 workers Saudi Arabia, Asir, Abha, Raydah, 18°12.265'N, 42°24.744'E, 2820 m; 1 worker Saudi Arabia, Asir, Abha, Raydah, 18°11.695'N, 42°23.818'E, 1897 m (all in KSMA) (*Al Dhafer* et al. *Leg.*); 1 worker in **NHMB**; 1 worker (casent0914585) and 1 queen (casent0914584) in **CASC** (*Al Dhafer et al. Leg.*).

#### Habitat.

**Figure 13.**
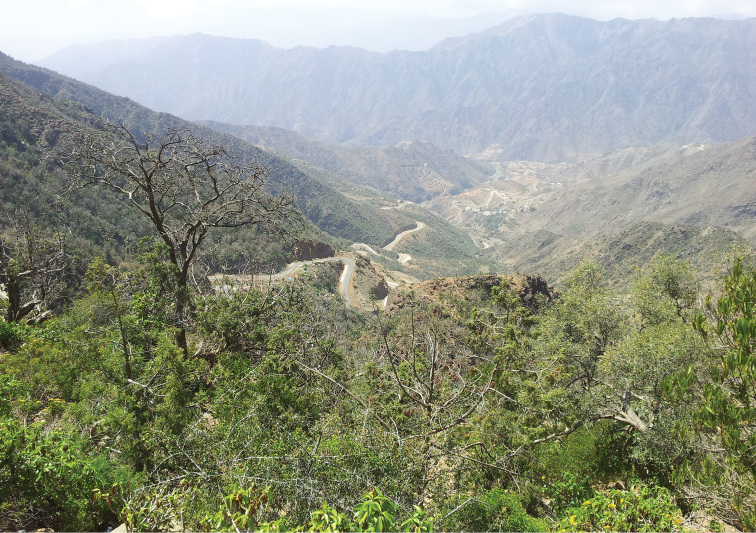
Raydah Protected area, habitat of *Tetramorium squaminode*. Photographer Mahmoud S. Abdel-Dayem.

*Tetramorium squaminode* was collected from Raydah Protectorate which includes one of the last remnants of dense juniper forest found on the KSA. The vegetation shows distinct altitudinal zonation, although there are variations within zones. The locality (Fig. 13) has a substantial high diversity of wild plants including: *Juniperus procera* Hochst. ex Endlicher (Cupressaceae), Olea europaea ssp. africana (Mill.) P. Green. (Oleaceae), *Maerua crassifolia* (Capparceae), *Panicum turgidum* (Poaceae), *Lycium shawii roem* (Solanaceae), *Haloxylon salicornicum* (Chenopodiaceae), *Aloe officinalis* Forssk. (Xanthorrhoeaceae), *Ziziphus spina-christi* (Rhamnaceae), *Opuntia ficus-indica* (L.) Mill. (Cactaceae), *Coffea arabica* (Rubiaceae) and *Acacia* spp. (Mimosaceae).

### Key to the Arabian species of the *Tetramorium squaminode*-group based on workers

**Table:** 

1	Strongly bicoloured species, head, mesosoma, petiole and postpetiole yellow, gaster brown to brownish yellow; eyes with ten ommatidia in the longest row; posterior margin of head concave; pronotal corners angled from above; posterior margin of head with hairs of more or less equal lengths; clubbed pilosity distributed over the entire body (Yemen & KSA)	***Tetramorium latinode* Collingwood & Agosti**
–	Weakly bicoloured species with cephalic dorsum and gaster dark brown, lateral head, mesosoma, petiole and postpetiole of lighter brown; eyes with 13 ommatidia in the longest row; posterior margin of head nearly straight; pronotal corners rounded from above; posterior margin of head with one pair of characteristically longer hairs on both corners; clubbed pilosity restricted to the gaster, few on mesosoma (KSA)	***Tetramorium squaminode* Santschi**

### Key to the Arabian species of the *Tetramorium squaminode*-group based on queens

**Table:** 

1	Strongly bicoloured, mesosoma, petiole, postpetiole, antennae and legs yellowish, head dorsum yellowish with scattered brownish tint, gaster brown; posterior margin of head distinctly concave; frontal carinae with only two pairs of hairs straddling the entire length, posterior corners of head without a single pair of longer hairs, instead about 8 subdecumbent hairs straddling the corners (Figs 4–6) (Yemen & KSA)	***Tetramorium latinode* Collingwood & Agosti**
–	Weakly bicoloured species with, head, mesosomal dorsum, petiole and postpetiole dark brown or blackish brown, gaster dark blackish brown to black, antennae and legs yellowish; posterior margin of head straight; frontal carinae with five pairs of distinctly long hairs straddling the entire length, posterior corners of head with some short hairs and a characteristic single pair of longer hairs (KSA)	***Tetramorium squaminode* Santschi**

## Discussion

Geologically, the Ethiopian and Arabian Peninsula mountains and highlands were separated approximately 13 my by the formation of the Great Rift Valley through a rifting process as the African continental crust separated (Davison et al. 1994, Bosworth et al. 2005). Therefore, it is not surprising that the faunal composition of the southwestern region of the Arabian Peninsula is similar to the Afrotropical region. Several works have confirmed the mentioned faunal similarity (Eig 1938; Zohary 1973; Bolton 1994, Lehrer and Abou-Zied 2008; Doha 2009; Aldawood et al. 2011; Sharaf and Aldawood 2011, 2012; Sharaf et al. 2012a, b, c; El-Hawagryi et al. 2013; Sharaf and Aldawood 2013; Sharaf et al. 2014). Consequently, the biogeography of *Tetramorium squaminode* in the southwestern Mountains of the Arabian Peninsula is obviously similar to that of East African specimens. *Tetramorium squaminode* represents a very characteristic Afromontane faunal element confined to localities at higher elevations above 2500 m. The East African records are all from high elevations above the forest line, such as Mt. Kenya at ca. 3200 m (F. Hita Garcia, pers. comm.), at different elevations from Mt. Kilimanjaro, Tanzania, including 3800 m (Santschi 1911) and 2600-2800 m (Santschi 1914), as well as another unknown site in Tanzania (Bolton 1980). In general, one can say that the faunal composition of the southwestern Mountains of the Arabian Peninsula is a combination of two major elements, Arabian endemic and Afrotropical elements. The *squaminode* group is a clear example of this since it has a single endemic species (*latinode*) and one widespread East African species (*squaminode*).

**Figure 14.**
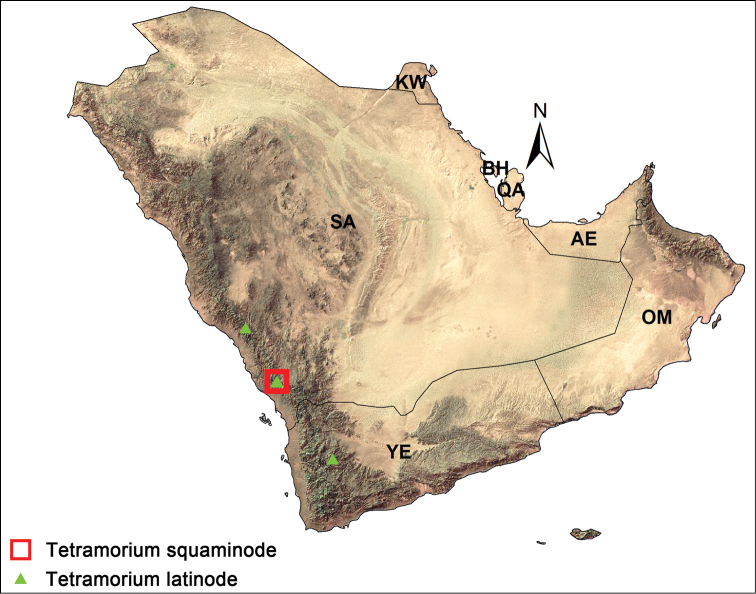
Distribution of *Tetramorium squaminode*-group in the Arabian Peninsula.

From another aspect, the regional distribution of the two species of the *Tetramorium squaminode*-group appears to be restricted to the southwestern Mountains of the Arabian Peninsula (Fig. 14). *Tetramorium latinode* was reported from Yemen and Al Sarawat Mountain, southwestern region of the KSA, whereas *Tetramorium squaminode* is only known from Asir Mountains. It is anticipated that the Arabian *Tetramorium squaminode*-group members have a broader distribution in the region than currently known, and will be found in additional localities at higher elevations, as well as in the Yemeni regions of these mountains. In addition, the record of *Tetramorium squaminode* in the Mountains of southwestern KSA, as well as other Afrotropical faunal elements recorded in the region, provides additional confirmations of the similarity of the entomofauna with the Afrotropical Region (Eig 1938; Zohary 1973; Bolton 1994, Lehrer and Abou-Zied 2008; Doha 2009; Aldawood et al. 2011; Sharaf and Aldawood 2011, 2012; Sharaf et al. 2012a, b, c; El-Hawagryi et al. 2013; Sharaf and Aldawood 2013; Sharaf et al. 2014).

## Supplementary Material

XML Treatment for Tetramorium latinode

XML Treatment for Tetramorium squaminode
